# The Influence of Electrode Design on Detecting the Effects of Ferric Ammonium Citrate (FAC) on Pre-Osteoblast through Electrical Cell-Substrate Impedance Sensing (ECIS)

**DOI:** 10.3390/bios13030322

**Published:** 2023-02-27

**Authors:** Zheyuan Zhang, Xichen Yuan, Huijie Guo, Peng Shang

**Affiliations:** 1 School of Life Sciences, Northwestern Polytechnical University, Xi’an 710072, China; 2 Key Laboratory for Space Biosciences and Biotechnology, Northwestern Polytechnical University, Xi’an 710072, China; 3 School of Mechanical Engineering, Northwestern Polytechnical University, Xi’an 710072, China; 4 Research & Development Institute, Northwestern Polytechnical University in Shenzhen, Shenzhen 518110, China

**Keywords:** ECIS, electrode design, sensitivity, osteoblast, dielectric properties

## Abstract

Detection sensitivity is a crucial factor in the application of ECIS sensors. For these biosensors, the electrode configuration has a direct impact on sensitivity, yet few studies on monopolar electrodes have been reported. In this study, ECIS sensor arrays, which have a series of working electrode configuration with a wide diameter range and different electrode number, were fabricated to monitor living osteoblast-like MC3T3-E1 cells. The experimental results revealed that when the electrode diameter was larger than 25 μm, electrodes with smaller diameter and number yielded higher impedance values and generated more impedance shift to cell status change. The membrane capacitance obtained by equivalent circuit fitting was at the same level. When the electrode diameter was even smaller, the results in detection of cell monolayer were opposite, and there was no distinct relationship between impedance and membrane capacitance shift to cell status change and electrode geometry. The proposed sensor chip, allowing for a sustained and stable detection of cellular impedance, provides the basis for the selection of the electrode configuration of monopolar electrodes. The test results of electrodes with a diameter of 25 μm and lower indicated the possibility of single cell impedance measurement, which can provide unique insight into the heterogeneous electrical behavior of cells, and, in this case, the electrode size should be close to the cell size.

## 1. Introduction

The electrical cell-substrate impedance sensing (ECIS) technique is a method monitoring cellular activities through weak electrical signals. When cells are added to the sensors and attach to the electrodes, they act as insulators. The electric field path formed by the excitation signal between the electrodes is impeded, and reflected in the response signal. When cells are stimulated to change their function, the accompanying changes in cell status alter the impedance [1,2]. Compared with fluorescence and optical methods [3], ECIS does not need extra cell labeling, and is considered as a non-invasive method for continuous cell assay. Since 1984 when Giaever and Keese first reported the modification of culture dishes with working and counter electrodes to monitor fibroblast behavior in tissue culture [4], the ECIS technique has been constantly developing and has been employed in different fields of cell biology research to study various cellular processes [5], including cell growth and proliferation [6,7], cell cycle [8], cell differentiation [9,10], cell motility and invasion [11,12] and cell signaling transduction [13,14]. Cell death can also be detected as the cells detach from the electrode, which makes ECIS become a promising technique for real-time screening of drugs [15,16,17].

In general, there are two types of cell impedance sensing electrodes. One consists of interdigitated electrodes: the working and counter electrode are the same in size, crossing together like the teeth of two combs [18]. The other type is made up of monopolar electrodes, which consist of a smaller working electrode and a much larger counter electrode. The smaller working electrode has larger current density and dominates the impedance change when the same density of cells are attached on both working and counter electrode [19].

At the beginning of the sensor design, the most concerning problem is the structure of the working electrode, because a different electrode layout directly affects the electrical response of the sensors and leads to different detection sensitivity. Detection sensitivity is a crucial criterion in the design and application of the sensors, which determines the quality and the accuracy of the measured data. For interdigitated electrodes, there have been numerous studies on the effects of electrode width, length and spacing on detection sensitivity [20]. However, fewer studies have been reported about the influence of electrode layout on the detection sensitivity of monopolar electrodes [21,22,23]. Zhang et al. found that working electrodes with a smaller radius generate more sensitive impedance responses to cell density change, and the distance between the edges of the sensing electrodes does not influence the measured impedance [22]. Montaño-Figueroa et al. reported that single electrodes are more suited for detecting cell motions, while multiple electrodes provide increased cell signal sensitivity when detecting dynamic cellular processes [23].

However, more research is still needed. Firstly, a study of the relationship between electrode diameter and sensitivity should be extended to a wider diameter range. For example, in single-cell based ECIS studies, the electrode dimension is reduced to cell size or even smaller, and the relationship between sensitivity and electrode diameter less than 50 μm is unclear. Secondly, the sensitivity of different electrodes in detecting changes in cell status due to chemical, biological or physical stimuli needs to be verified. In addition, only impedance data measured from different electrodes were analyzed in these studies, and further analysis of cell dielectric properties is needed. In view of the above problems, we designed and fabricated ECIS sensor arrays which have a series of working electrode configuration with a wide diameter range to monitor living osteoblast-like MC3T3-E1 cells. Then, an exogenous iron preparation, ferric ammonium citrate (FAC) was used to change the cell status, and the sensitivity of different electrodes in measuring FAC-induced impedance change was analyzed. Finally, an electrical equivalent circuit was proposed to model the changes of dielectric properties of cells treated by FAC.

## 2. Materials and Methods

### 2.1. Design and Fabrication Process of the Sensor Chip

In order to clarify the influence of electrode design on the test performance of a cell impedance sensor chip, the microelectrode arrays composed of circular electrodes with a combination of different diameters and numbers are designed. The electrode diameter ranges from 10 μm to 250 μm. These different electrodes are arranged on two sets of sensor chips. The electrode with larger diameters (50–250 μm) were arranged together and named type-A. The electrodes with smaller diameters (10–25 μm) were arranged in type-B sensors. All electrode dimensions are listed in Table 1. Because the commercial cell impedance sensors (Applied Biophysics, Troy, NY, USA) have an electrode diameter of 250 μm, we also fabricated sensor chips with the electrode dimension of D250N6 represented in Table 1, named type-C.

**Figure 1:**
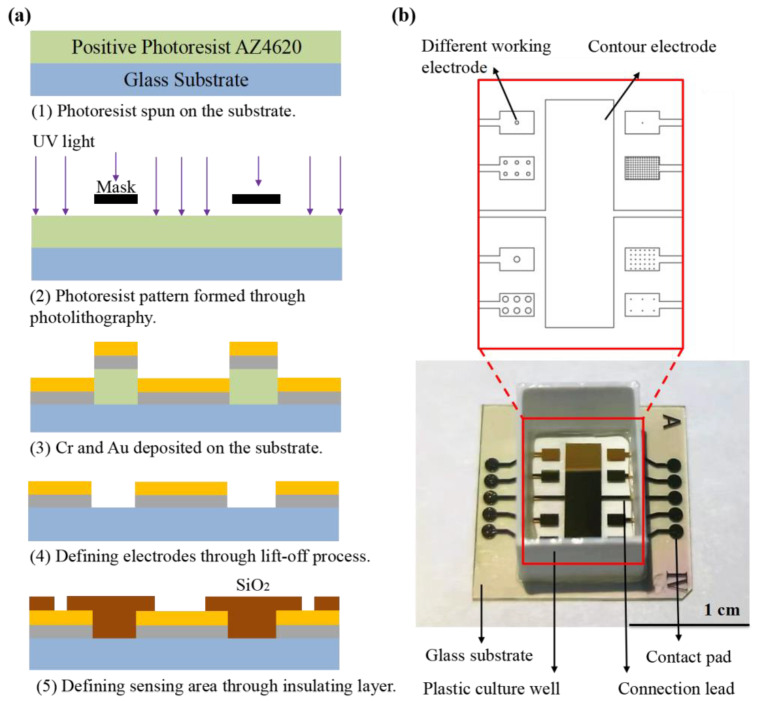
(**a**) Schematic representation of ECIS sensor fabrication, including (1) photoresist coating preparation; (2) photolithography; (3–4) evaporation of chromium and gold layer on glass substrate and patterned by life-off process; (5) growth of insulating layer made of SiO_2_ by PECVD and patterning of the insulating layers by gas etching technique. (**b**) Picture of ECIS sensor arrays.

Figure 1a describes the fabrication process for the proposed sensor chip. The sensor arrays were fabricated on glass substrate with a thickness of 1 mm. The standard lift-off photolithography process was used to pattern the electrode using positive photoresist AZ4620. Metal films of chromium (10 nm) and gold (100 nm) were evaporated onto the substrate to form the electrodes. After the lift-off process with acetone, a layer of silicon oxide (SiO_2_) was deposited by plasma-enhanced chemical vapor deposition (PECVD) covering the entire substrate as an insulating layer. Then a second photolithography was performed to expose the working electrode, counter electrode and contact pads by etching the SiO_2_ to the gold layer using Gas Plasma Etcher PlasmaPro 100 Polaris ICP RIE (Oxford, London, UK). Finally, the slide was cleaned by ultrasonic cleaning in acetone, ethanol and deionized water, and then dried under nitrogen flow. Before testing, a 3D-printed plastic well made of polylactic acid (with the size of 8 × 10 × 14 mm) was glued onto sensor arrays using biocompatible glue as the culture well (Figure 1b).

### 2.2. Experimental System Setup

**Figure 2:**
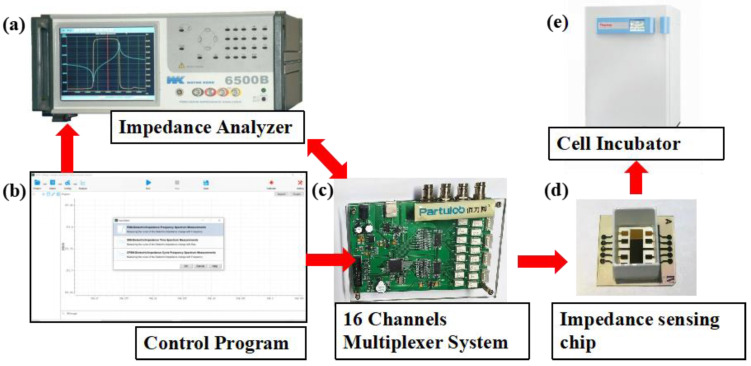
Experimental setup for cell impedance measurement. Impedance measurement and data acquisition were accomplished by impedance analyzer (**a**) and a 16 channel multiplexer (**c**), which were controlled by a computer program (**b**). The sensor arrays seeded with cells (**d**) were kept inside the CO_2_ incubator (**e**).

Electrical impedance spectroscopy was measured by Wayne Kerr Electronics 1J65120B impedance analyzer (Wayne Kerr Electronics, West Sussex, UK). Continuous multichannel data acquisition was accomplished by a 16 channel multiplexer between the impedance analyzer and the sensor arrays. The impedance analyzer was connected to a computer through a GPIB card and controlled with computer programs. The sensor arrays seeded with cells were kept inside the incubator at 37 °C with 5% CO_2_ during the impedance measurement. The experimental setup is shown in Figure 2.

### 2.3. Cell Culture Protocol

Murine osteoblastic cell line MC3T3-E1 Subclone 4 [24] used in this study was provided by Prof. Hong Zhou of the University of Sydney [25]. The cells were maintained by α-Minimum Essential Medium (Gibco, Grand Island, NY, USA) supplemented with 10% fetal bovine serum (Gibco, Grand Island, NY, USA), 1% penicillin-streptomycin and 2 mM L-glutamine, in a humidified 5% CO_2_ incubator at 37 °C. Before the experiment, the sensors were sterilized with 75% ethanol for 5 min, dried with nitrogen flow and irradiated with ultraviolet for 1 h. After that, culture medium was added into the sensors, and the sensors were placed in the incubator for the absorption of ions and proteins on the electrode surfaces for 10 min. The volume of culture medium added to each sensor was 500 μL. Cells were seeded uniformly with the density of 1 × 10^5^ cells/cm^2^ or 5 × 10^4^ cells/cm^2^. No pre-treatment of the sensor with adhesive support such as extracellular matrix was applied for the cells.

### 2.4. Impedance Measurement and Data Processing

Electrical impedance spectrum was measured in the frequency range of 1 kHz to 1 MHz, using an AC voltage of 25 mV amplitude with no DC bias. Before the measurement, a standard calibration procedure was conducted using a series of precision resistors and capacitors. The complex impedance is obtained using:

Z = Z_RE_ + jZ_IM_ (1)

where Z_RE_ and Z_IM_ are the real and imaginary part of the complex impedance, and j is the imaginary number. The magnitude of the impedance was calculated using:

(2) $$│Z│=\sqrt{{\left(Z_{\mathrm{RE}}\right)}^{2}+{\left(Z_{\mathrm{IM}}\right)}^{2}}$$

To analyze the cellular impedance, the impedance of the electrode–electrolyte interface and culture medium, which is not relevant to that of the cells, was first monitored after incubation with culture medium only, expressed as Z_0_(f, t_0_). The total impedance with cells added on the sensor is expressed as Z(f, t), where f is the measuring frequency, and t is the time after cell seeding. The influence of cells on the measured impedance is defined as:

Z_cell_(f, t) = Z (f, t) − Z0(f, t_0_), (3)

Normalized impedance (NI) = Z_cell_(f, t)/Z_0_(f, t_0_), (4)

**Figure 3:**
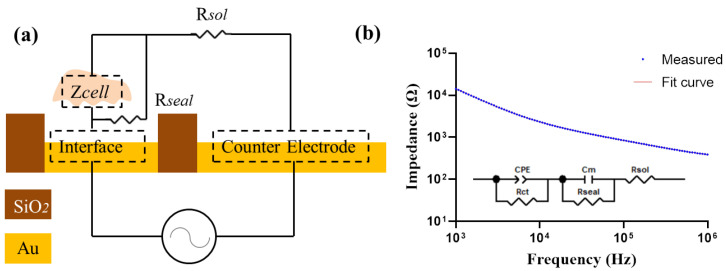
(**a**) Simplified circuit model for cell impedance measurement. (**b**) The measured impedance spectra with cells on the electrode. An equivalent electrical circuit model is used to fit the measured data.

Furthermore, to determine the cellular electrical property values from the measured data, a simplified circuit model of the cell–electrode structure was constructed as shown in Figure 3. Z_solution_ was expressed as a pure resistance R_sol_. As cells grow, they can secrete metabolites to the culture medium to increase its conductivity. Therefore, fresh medium was carefully replaced before each measurement. Z_interface_ was assumed as Z_CPE_ in parallel with a charge transfer resistance R_ct_ [26]. The constant phase element CPE is used to characterize the electrical double-layer impedance in the electrode–electrolyte interface due to the inhomogeneities on the surface of the electrode [27]. It can be expressed as:

ZCPE = 1/[Q(jω)n](5)

where ω is the angular frequency and n is a constant (0 ≤ n ≤ 1), Q is the magnitude of Z_CPE_ and j is the imaginary number. In the proposed sensor design, the area of the working electrode is much smaller compared with that of the counter electrode (30 mm^2^), so the surface interface impedance of the counter electrode is negligible.

The cellular impedance was built as a membrane capacitance C_m_ [28,29,30]. In addition, a gap between cell membrane and the electrode surface is formed due to the finite binding of cells to the surface, which was considered as in parallel with the cells as R_seal_ [31]. By fitting the measured impedance spectrum into the circuit model, the values of each electronic element are obtained. The fitting was accomplished by the ZView software, version 3.1.

## 3. Results and Discussion

### 3.1. The Impedance Response from the Fabricated Sensor Arrays

**Figure 4:**
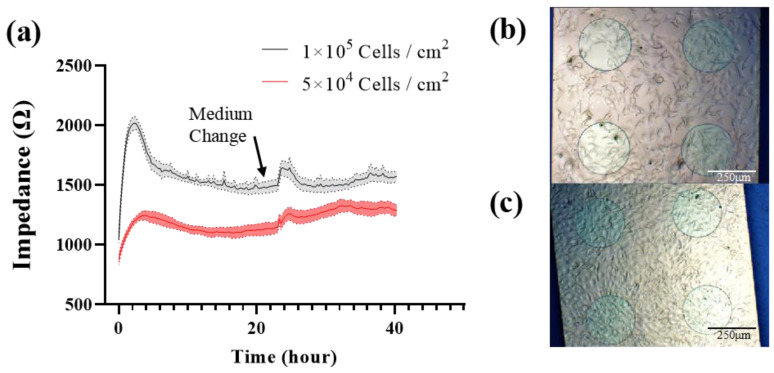
(**a**) 40 h continuous impedance measurement of MC3T3-E1 cells with different seeding density at 10 kHz. Cell image was taken before media change. (**b**) 5 × 10^4^ Cells/cm^2^. (**c**) 1 × 10^5^ Cells/cm^2^. Results are shown as means ± standard error (SE) (n = 4).

MC3T3-E1 cells were seeded onto the type-C sensor at 1 × 10^5^ cells/cm^2^ (group A) and 5 × 10^4^ cells/cm^2^ (group B), respectively. The impedance was measured in real time every 15 min at 10 kHz. At 23 h, fresh medium was exchanged. Figure 4 shows the cell impedance response and morphology. The impedance was significantly higher in group A than in group B to distinguish the different seeding densities. After seeding, the impedance was increased rapidly in the first 4 h in both groups, which indicated cell adherence. After that the impedance began to decline, due to the morphology change from the initial round or oval shape to the elongated spindle shape. After a medium change, the impedance was increased because of the lower conductivity of the fresh medium. According to the imaging information (Figure 4b,c), the seeding density of group A was sufficient to form the cell monolayers, and the cells were growing with contact inhibition. After the change to a fresh medium, cells released metabolites to reduce the conductivity of the medium, but did not grow as fast as group B, so the impedance of group A returned to their previous levels, while the impedance of group B continued to increase following cell growth.

According to the above results, the impedance-based biosensors have been successfully developed for discriminating cell numbers and monitoring cellular activities. We chose the seeding density of 1 × 10^5^ cells/cm^2^, which allows the cells to form monolayers to completely cover the electrode theoretically. This reduced the measurement variance due to a different area of the cells covering the electrode, which ensured that the difference in impedance measurement results is due to the electrode structure only.

### 3.2. Impact of Electrode Design on Detecting Cell Monolayer

**Figure 5:**
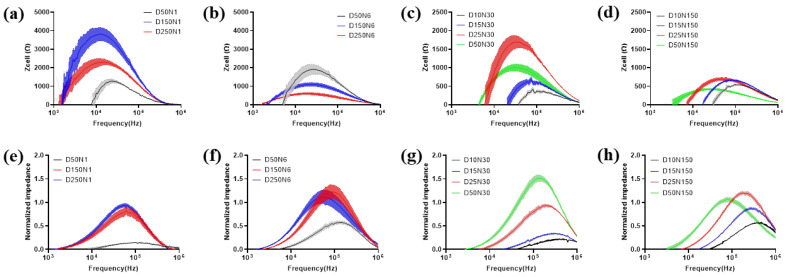
Electrode design impact on measurement of MC3T3-E1 cell monolayer. The data is presented as Z_cell_ (**a**–**d**) and NI (**e**–**h**). For comparison, the electrode number is kept fixed on 1 (**a**,**e**), 6 (**b**,**f**), 30 (**c**,**g**) and 150 (**d**,**h**). Results are shown as means ± SE (n = 4).

MC3T3-E1 cells were seeded onto type-A and type-B sensors at 1 × 10^5^ cells/cm^2^, and the cells were incubated for 24 h to reach the steady state. After that, impedance measurements were conducted by each type of electrode and displayed as Z_cell_ and NI (Figure 5). The test results obtained on the cells seeded at the same density and incubated in the same environment varied significantly by using different types of electrodes. For the electrodes in type-A sensors, Z_cell_ is higher for the electrode with a lower diameter and lower electrode numbers, except for the D50N1 electrode (Figure 5a,b). However, NI presented an approximately opposite trend (Figure 5e,f). For the electrodes in type-B sensors, when the electrode diameter was larger than 25 μm, the test results of both Z_cell_ and NI followed the same trends with the electrode in type-A sensors, except for NI of D50N150. When the electrode diameters were even smaller, the test values became lower and decreased with the diameter (Figure 5c,d,g,h). 

The effects of electrode geometry on cell impedance measurement had a critical value of 25 μm, which is close to the dimension of MC3T3-E1 cells. When the electrode area was large enough, electrodes with lower diameter and number provide less current paths, which increased the impedance correspondingly. Meanwhile, the electrode area was large enough to guarantee an adequate number of cells for measurement, so the influence of cell coverage on overall impedance was greater. However, when the working electrode area was even smaller than the cell, the cell contact area was insufficient for measurement. As a result, the cellular impedance contributed less to the overall impedance. For NI, although the smaller working electrode generates higher Z_cell_, the impedance of the electrode–electrolyte interface Z_0_ was much higher, so the data normalized with Z_0_ decreased accordingly. 

Notably, the optimal frequency, which allows obtaining the largest difference with and without cells for analysis varied in both Z_cell_ and NI with a different electrode pattern. In general, the optimal frequency of NI was higher than that in Z_cell_ for the same electrode pattern. The optimal frequency for both Z_cell_ and NI was increased with a decreased electrode diameter. The maximum values of Z_cell_ and NI measured by various electrode patterns, and the corresponding optimal frequency, are exhibited in Supplementary Table S1.

### 3.3. Electrical Evaluation of the Effects of FAC on MC3T3-E1 Cells

#### 3.3.1. Effects of FAC on the Impedance of MC3T3-E1 Cells

**Figure 6:**
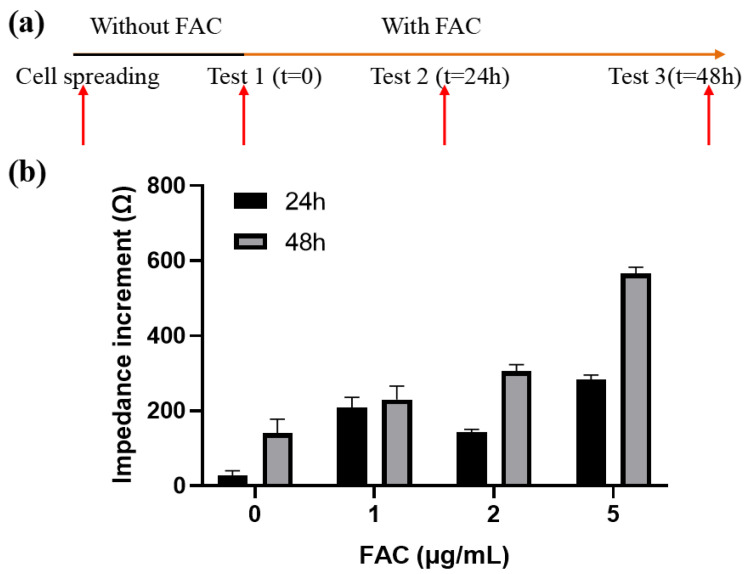
Effects of FAC on the impedance of MC3T3-E1 cells. (**a**) Schematic diagram of the experimental process. (**b**) Impedance increments of cells treated by different concentration of FAC. Results are shown as means ± SE (n = 4).

From the above results we can conclude that electrodes with different geometry have different detection performance on the cell monolayer. The next step is to determine the detection capability of various electrode patterns on cells under a different status. To induce changes in cell growth behavior, an iron preparation, ammonium ferric citrate (FAC), which increases the viability of MC3T3-E1 cells with a suitable concentration range (Supplementary Figure S1), was introduced in the culture medium to affect cell growth. MC3T3-E1 cells were firstly seeded at 1 × 10^5^ cells/cm^2^ and incubated overnight for 8 h, and then different concentrations of FAC (with the mass concentration of iron element of 0, 1, 2, 5 μg/mL) were added. Impedance spectrum measurements were conducted after 24 and 48 h. The medium was replaced before each measurement to eliminate the influence of the additional FAC and extracellular metabolic product on the conductivity of the medium, so the impedance change was only affected by the state of the cells on the electrode, theoretically. The procedure was shown in Figure 6a. Impedance data was analyzed at the optimal frequency of 18 kHz. Z_cell_ measured at the beginning and afterwards were denoted as Z_0_ and Z_t_, respectively. The impedance increment was represented as ΔZ = Z_t_ − Z_0_.

In general, the cellular impedance increased with time. After 24 h and 48 h of incubation, the cellular impedance without FAC addition increased (36.14 ± 11.4) Ω and (174.36 ± 18.61) Ω, respectively. When FAC was added, the cellular impedance increased even more and had a positive relationship with the FAC concentration within the given range (Figure 6b). The impedance increment of 5 μg/mL FAC group after 48 h was the largest, reached up to (565.83 ± 17.22) Ω, which is about triple the number from the 0 μg/mL group. The reason for the impedance changes was on the one hand the presence of the cells on the surface of the electrode. Although the cells formed a monolayer on the electrode, they continued to grow and divide, further occupying the uncovered area on the electrode. On the other hand, FAC could affect the dielectric properties of the cell membrane, which also contribute to the impedance change. This part will be discussed later.

#### 3.3.2. FAC-Induced Impedance Change Detected by Different Types of Electrodes

Next, we analyzed the detection capability of various electrode patterns on FAC-induced cellular impedance shift. MC3T3-E1 cells were seeded in different types of sensors, the following experimental procedures were the same as Figure 6a described. Specifically, the sensors were divided into two groups with FAC addition of 0 μg/mL and 5 μg/mL. The impedance data collected from different electrodes were analyzed at their respective optimal frequency, shown in Supplementary Table S1. The cellular impedance increment was represented as Z_t_ − Z_0_. Impedance change in cells with no addition of FAC was presented as ΔZ_A_, otherwise as ΔZ_B_, respectively.

**Figure 7:**
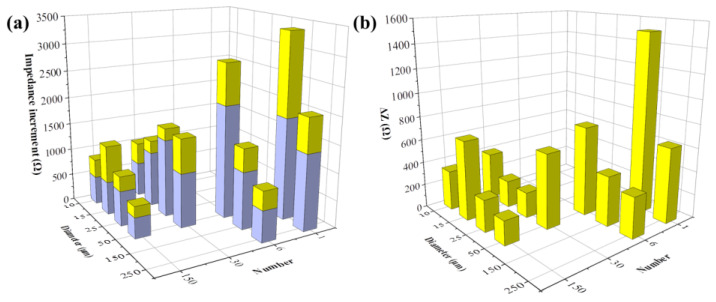
FAC-induced impedance change detected by different types of electrodes. (**a**) Impedance increment of MC3T3-E1 cells treated by 0 μg/mL (blue column, ΔZ_A_) and 5 μg/mL FAC (the whole column, ΔZ_B_); (**b**) Additional impedance increments induced by 5 μg/mL FAC (ΔZ = ΔZ_B_ − ΔZ_A_).

Figure 7 illustrates the cellular impedance increment after 48 h of incubation. ΔZ_A_ was displayed with blue columns. All types of electrodes have detected the additional impedance increment in FAC-treated groups. The extra increment of cell impedance caused by FAC addition (ΔZ = ΔZ_B_ − ΔZ_A_) is shown with the yellow columns on top of the blue columns (Figure 7a), or exhibited independently in Figure 7b. ΔZ_A_ showed the cellular impedance change under normal cell growth over time, which was not affected by the addition of FAC. The detection performance on ΔZ_A_ varied in different electrodes. As Figure 7a illustrates, ΔZ_A_ increased with the decreased electrode number. However, the effects of diameter size were different. When the electrode diameter was larger than 25 μm, ΔZ_A_ decreased, while with the increased diameter, when the electrode diameter was smaller than 25 μm, ΔZ_A_ changed in an opposite way. The cell growth rate across different locations (i.e., different places on the electrodes) was theoretically consistent, so the different ΔZ_A_ values indicated the differences in electrode test performance only. Moreover, the change rules of ΔZ_A_ with electrode patterns were the same as Z_cell_, illustrated in Figure 5a–d, indicating that the electrode pattern more sensitive to the presence of cell monolayer also generates more impedance shift to cell density change.

The additional impedance increments caused by FAC, presented by ΔZ = ΔZ_B_ − ΔZ_A_, are illustrated in Figure 7b. When the electrode diameter was larger than 25 μm, the change rule with ΔZ was consistent with ΔZ_A_, indicating the change rules on test performance of different electrode pattern also applied to the cellular impedance change induced by external factors such as addition of FAC. However, when the electrode diameter was smaller, there was no distinct relationship between ΔZ change and the electrode diameter/numbers. The possible reason could be that there were fewer cells on the small electrodes, especially when the electrode diameter was lower than the dimension of MC3T3-E1 cells, and the low number of examined cells was not enough to eliminate the cell heterogeneity.

#### 3.3.3. Effects of FAC on Membrane Capacitance of MC3T3-E1 Cells

To determine the cellular electrical property values from the measured data, we analyzed simulated values of the cellular membrane capacitance in the equivalent circuit model illustrated in Figure 3a. The cellular impedance data measured by electrode D250N6 and the fitted curve were almost identical (Figure 3b), and the fitting error of different elements were less than 5%. The fitting results were also good in all other kinds of electrodes, shown in Supplementary Figure S2.

**Figure 8:**
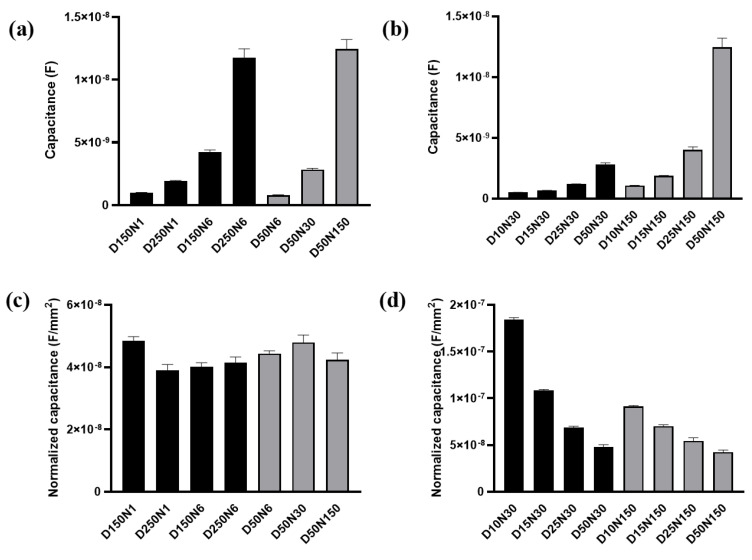
The membrane capacitance of MC3T3-E1 cells acquired from the equivalent circuit model. (**a**,**b**) The membrane capacitance obtained by fitting from the test results of different electrodes. (**c**,**d**) The membrane capacitance normalized by the total area of working electrode. Results are shown as means ± SE (n = 4).

The membrane capacitance C_m_ was firstly analyzed with the data measured at t_0_ before the FAC was added (Figure 6a). The C_m_ values obtained from different electrode types were shown in Figure 8a,b. A significant positive correlation of C_m_ and the working electrode area can be observed. The larger working electrode can detect a greater number of cells. The membrane capacitance of each cell on the electrode is regarded as in parallel. More cells under detection means more membrane capacitance unit in parallel in the circuit, and the total C_m_ increased accordingly. After noticing this phenomenon, we used the working electrode area to normalize the C_m_ value (Figure 8c,d). The normalized C_m_ was also related to electrode diameter. When the electrode diameter was larger than 50 μm, the normalized C_m_ obtained from different electrodes remained at the same level and had no significant differences regardless of electrode diameter or number (Figure 8c). The cells were regarded as evenly distributed across the sensing area. When the electrode diameter was large enough, the number of cells per unit electrode area was the same. The intrinsic membrane capacitance of the untreated cell on different electrodes had no significant differences, so the normalized C_m_ remained at the same level. However, when the electrode area was smaller, the normalized C_m_ was increased with the decreased electrode diameter/number (Figure 8d).

**Figure 9:**
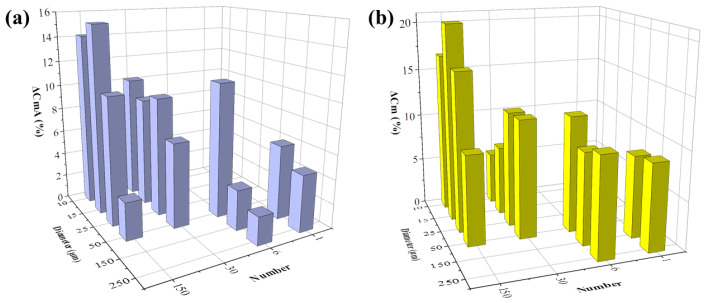
FAC-induced membrane capacitance change detected by different types of electrodes. (**a**) Membrane capacitance increment of MC3T3-E1 cells treated by 0 μg/mL (blue column, ΔC_m_A). (**b**) Membrane decrease induced by 5 μg/mL FAC (ΔC_m_ = ΔC_m_A − ΔC_m_B).

The C_m_ change in percentage after 48 h of cell growth with or without FAC addition was then analyzed. Capacitance change in cells with no addition of FAC was presented as ΔC_m_A, otherwise as ΔC_m_B, respectively. The ΔC_m_A increment during normal cell growth over time also varied in different electrode types. When the electrode diameter was larger than 25 μm, ΔC_m_A was increased with decreased electrode number and diameter (Figure 9a). This change rule was consistent with the impedance increment (ΔZ_A_) illustrated in Figure 7a. The consistency of the two parameters detected by different types of electrode can be attributed to cell growth and division. The increased number of cells on the electrode caused increased impedance, and it also means more membrane capacitance in parallel to increase the total capacitance. The smaller electrode could detect higher C_m_ change, but the capacitance value seemed to be random among the different electrode designs.

However, after FAC addition, although cells grew normally and increased in number, ΔC_m_B detected by all kinds of electrode decreased, which indicated changes in the intrinsic electrical properties of the cell. The FAC-induced capacitance decrease was calculated by ΔC_m_ = ΔC_m_A − ΔC_m_B, illustrated in Figure 9b. ΔC_m_ remained at the same level of about 10% when the electrode diameter was 50 μm or larger, but when the electrode diameter was smaller, ΔC_m_ was random. The consistency of ΔC_m_ detected by larger electrodes could be attributed to the average membrane capacitance changes treated by FAC in a relatively large number of cells. When the electrode diameter was smaller, fewer cells were detected and the heterogeneity of the cells caused fluctuation of the data. The changes of cell membrane capacitance also contributed to the cellular impedance change. The cell membrane is insulating at low frequency band, and contributes to impedance change at medium and high frequency [32], which involved the optimal frequencies of different types of electrodes used in the experiment, ranging from 10 kHz − 100 kHz. According to the simulation results reported by Ren and Chui [32], when the membrane capacitance is reduced while the other factors are kept fixed, the impedance of adhered cells is larger. This phenomenon is consistent with our results that FAC-induced membrane capacitance decrease contributed to the overall cellular impedance increment.

The FAC of 1–5 μg/mL used in the experiments increased viability of MC3T3-E1 cells (Supplementary Figure S1), however, with the limitation of the optical imaging, information on the number or area of cells covered on the electrode was not available and the capacitance normalized by the electrode area could not reflect the membrane capacitance of each cell. Hence, the FAC-induced cellular impedance increment could be caused by the increased cell number as well as the decreased membrane capacitance, but their respective contributions could not be quantified. Further quantification of the impedance and capacitance of each cell could rely on fluorescence imaging of fluorescent protein labeled cells or nuclear staining to obtain the specific number of cells growing on the electrode.

## 4. Conclusions and Prospects

We have successfully presented an impedance-based biosensor for monitoring cellular activities of MC3T3-E1 cells. The experimental results reveal how sensor dimension influences the detection functionality. When the electrode diameter is larger than 25 μm, electrodes with fewer number and smaller diameter generate more impedance response to cell monolayer and cell density change. The electrodes with diameter smaller than 25 μm generate opposite results. The FAC-induced cellular impedance increment detected by an electrode larger than 25 μm has the same change rule to the detection of cell density change. However, there is no distinct relationship between impedance increment and electrode geometry in the smaller electrode. The equivalent circuit model indicates the cellular activities can be identified by the electrical parameters in the circuit. The FAC-induced capacitance decrease detected by larger electrode remains at the same level but seems to be random in the smaller electrode.

In summary, this work provides the basis for the selection of the electrode configuration of monopolar electrodes. The electrode design should be application specific, considered according to the researchers’ requirements of cell size and number of cells for measurements. When large number of cells are detected, a relatively smaller electrode diameter can generate higher impedance values to increase signal-to-noise ratio and improve data quality. The test results of electrodes with diameter of 25 μm and lower indicated the possibility of single cell measurement. By integrating with microfluidic technology, this type of biosensor could be used as single cell heterogeneity detection. Meanwhile, this type of biosensor could also help us to distinguish different substances and their concentration changes through the variation of cell resistance and cell capacitance.

## Figures and Tables

**Table 1:** Configuration of ECIS sensors.

Sensor Type	Electrode Type	Working Electrode Area (μm)^2^
Type A	D50N1	1963.5
	D50N6	11,781.0
	D50N30	58,904.9
	D50N150	294,524.3
	D150N1	17,671.5
	D250N1	49,087.4
	D150N6	106,028.8
Type B	D250N6	294,524.3
	D10N30	2356.2
	D15N30	5301.3
	D25N30	14,726.2
	D10N150	11,781.0
	D15N150	26,507.2
	D25N150	73,631.1
Type C	D250N6	294,524.3

## Data Availability

Original data from this study is held by the author Zheyuan Zhang and can be obtained by request at zzy19960319@mail.nwpu.edu.cn.

## Supplementary Materials

The following supporting information can be downloaded at: https://www.mdpi.com/article/10.3390/bios13030322/s1, Figure S1: MC3T3-E1 cell viability was detected by Cell counting kit-8 in treatment with different concentrations of FAC at (a) 24 h, (b) 48 h. Data was shown in means ± SD (n = 5); Figure S2: Impedance data measured by different kinds of electrode (marked by dot) and the fit curve using the equivalent circuit model; Figure S3: Microscope image of capture probes modified magnetic beads for capturing CEM cells. Table S1: The maximum detection value of Zcell and NI, as well as their responsive optimal frequencies in different electrode designs.
