# Supplementary material for: The Influence of Electrode Design on Detecting the Effects of Ferric Ammonium Citrate (FAC) on Pre-Osteoblast through Electrical Cell-Substrate Impedance Sensing (ECIS)
Source: Biosensors (Basel). 2023 Feb 27;13(3):322. doi: 10.3390/bios13030322 (PMC10046662; doi:10.3390/bios13030322)
Supplement: Supplementary file 1 [file biosensors-13-00322-s001.zip › biosensors-2209919-supplementary.pdf]

Supporting information

The Influence of Electrode Design on Detecting the Effects of Ferric Ammonium Citrate (FAC) on Pre-Osteoblast through Electrical Cell-Substrate Impedance Sensing (ECIS)

Zheyuan Zhang [1,2,†], Xichen Yuan [2,3,†], Huijie Guo [1,2] and Peng Shang [2,4,*]

Table S1. The maximum detection value of Zcell and NI, as well as their responsive optimal frequencies in different electrode designs.

Electrode type	Maximum detection value (Zcell)	Optimal Frequency	Maximum detection value (NI)	Optimal Frequency
D50N1	1277.7	23101.3	0.1478	100000
D150N1	3820.7	11497.6	0.8286	61359.1
D250N1	2304.7	14174.7	0.9393	57223.7
D50N6	1919.3	26560.9	0.5759	141747.4
D150N6	1100.2	23101.3	1.2286	81113.1
D250N6	604.3	18738.2	1.1165	57223.7
D10N30	419.0	81113.1	0.2220	403701.7
D15N30	684.5	75646.3	0.3374	305385.6
D25N30	1696.0	35111.9	0.93111	200923.3
D50N30	1005.0	30538.6	1.51259	123284.7
D10N150	541.9	93260.3	0.57368	464158.9
D15N150	669.2	86974.9	0.87233	265608.8
D25N150	696.8	49770.2	1.19912	174752.8
D50N150	421.0	26560.9	1.06160	81113.1

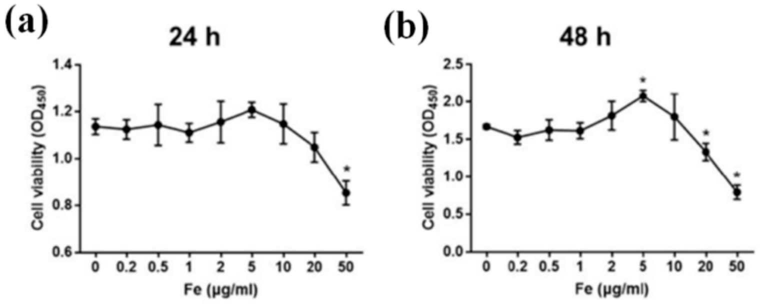

Figure S1. MC3T3-E1 cell viability was detected by Cell counting kit-8 in treatment with different concentrations of FAC at (a) 24h, (b) 48h. Data was shown in means ± SD (n = 5).

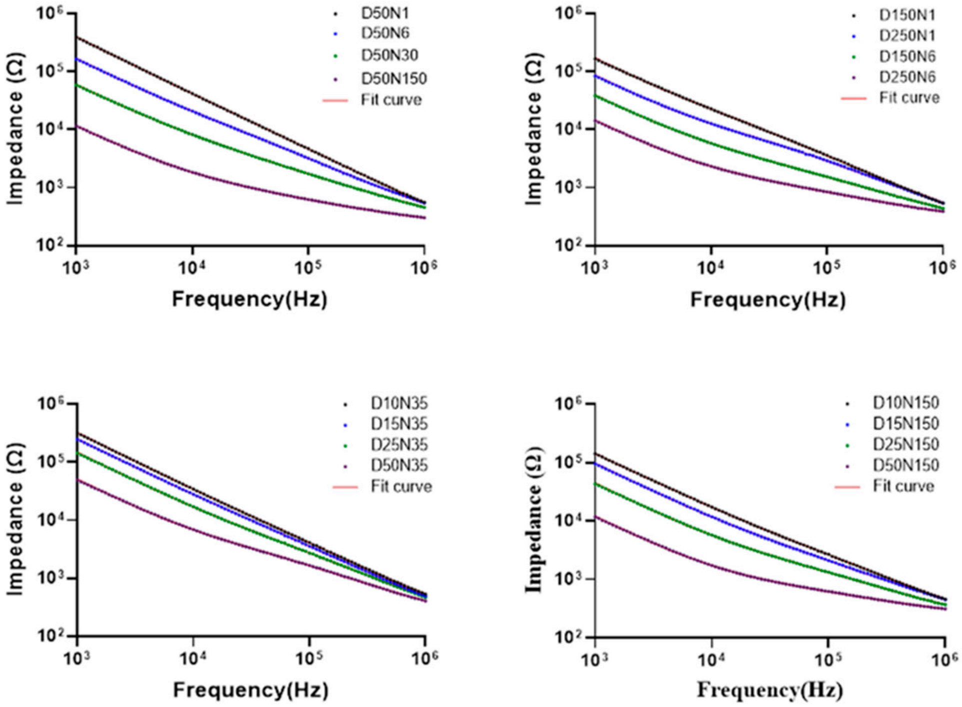

Figure S2. Impedance data measured by different kinds of electrode (marked by dot) and the fit curve using the equivalent circuit model.

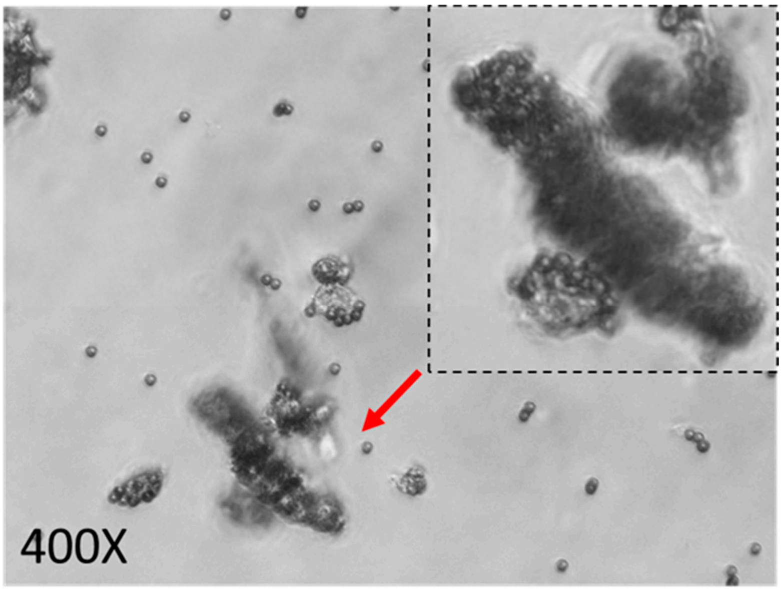

Figure S3. Microscope image of capture probes modified magnetic beads for capturing CEM cells.

$$\text{modified ratio} = \frac{A_0 - A_1}{A_0} \times 100\%$$

A_0 was the UV absorption at 260 nm before modification,
A_1 was the UV absorption at 260 nm after modification.
